# Association between pre- and postoperative rotational mismatches of the femorotibial components and bones in bi-cruciate retaining and posterior stabilized total knee arthroplasty

**DOI:** 10.1038/s41598-023-42243-6

**Published:** 2023-09-09

**Authors:** Shine Tone, Masahiro Hasegawa, Yohei Naito, Hiroki Wakabayashi, Akihiro Sudo

**Affiliations:** Department of Orthopaedic Surgery, Mie University, Graduate School of Medicine, 2-174 Edobashi, Tsu, Mie 514-8507 Japan

**Keywords:** Medical research, Outcomes research

## Abstract

To clarify the association between pre- and postoperative rotational mismatches of the femorotibial components and bones for total knee arthroplasty (TKA) with bi-cruciate retaining (BCR) design and with fixed bearing posterior stabilized (PS) design. This retrospective cohort study included 40 BCR TKAs and 50 PS TKAs. Pre- and postoperative rotational mismatches of the femorotibial components and bones were measured by three-dimensional assessment based on computed tomography imaging. The mean value and percentage within ± 5° of pre- and postoperative rotational mismatches were compared between BCR TKA and PS TKA. Correlations between pre- and postoperative rotational mismatches of the femorotibial components and bones were investigated in BCR TKA and PS TKA. There was no significant difference in mean preoperative rotational mismatch of femorotibial components and bones between BCR TKA and PS TKA. Mean postoperative rotational mismatch of femorotibial components and bones was significantly greater in BCR TKA than in PS TKA. Postoperative rotational mismatch of the femorotibial components was within ± 5° in 21 knees (52.5%) for BCR TKA and in 43 knees (86.0%) for PS TKA. The rate of postoperative rotational mismatch of the femorotibial components and bones within ± 5° was significantly lower for BCR TKA than for PS TKA. In BCR TKA, there was a positive correlation between pre- and postoperative rotational mismatches of the femorotibial components and of bones. We consider these results can be attributed to the retention of both cruciate ligaments, which may affect the reduction of rotational permittance of the components and bones.

## Introduction

Rotational alignment of components is an important factor for successful total knee arthroplasty (TKA), and malalignment is associated with knee pain^1–4^, abnormal gait patterns^5^, severe knee stiffness^6–8^, inferior outcome^1^, and early revision arthroplasty^9, 10^. Several studies have reported rotational mismatch between the femoral and tibial components and also between the femur and tibia in TKA^11, 12^. In contrast, few studies have investigated rotational alignment in TKA with bi-cruciate retaining (BCR) design^13^. BCR TKA is an alternative technique to conventional TKA that incorporates cruciate-retaining and posterior-stabilized (PS) design^14^. The most important difference between BCR TKA and conventional TKA is the preservation of both cruciate ligaments, which is important in terms of restoring joint kinematics to near normal and posterior femoral rollback, reproducing medial pivot rotation, and preserving normal proprioception^15–18^.

We hypothesized that there might be differences between BCR TKA and PS TKA regarding the association between pre- and postoperative rotational mismatches of the femorotibial components and bones; however, no previous study has investigated this association. Therefore, the aim of this study was to clarify the association between pre- and postoperative rotational mismatches of the femorotibial components and bones in BCR TKA and in fixed-bearing PS TKA.

## Materials and methods

### Patient selection

Included in the study were patients who underwent primary TKA at our institution between February 2016 and February 2019. Among these patients, 43 TKAs were performed using the BCR design (Vanguard XP Total Knee System; Biomet, Warsaw, IN), 112 TKAs were performed using the fixed-bearing PS design (Vega Knee System; B. Braun Aesculap, Tuttlingen, Germany), and 18 TKAs were performed using other designs. The inclusion criteria for BCR TKA were knees with primary various osteoarthritis and with both cruciate ligaments intact and functional. The retention of both cruciate ligaments was confirmed by magnetic resonance imaging and visualized intraoperatively. The exclusion criteria were knees with rheumatoid arthritis, previous surgery, flexion contracture ≥ 20°, valgus deformity, and lack of pre- or postoperative computed tomography (CT) images. Ultimately, 40 BCR TKAs and 50 PS TKAs were included in this study. We recorded patient demographics including sex, age, body mass index, maximum flexion angle, maximum extension angle, and hip-knee-ankle angle. All preoperative and postoperative evaluations were performed by the author (ST) and all operations were performed by a senior surgeon (MH). All patients underwent pre- and postoperative CT imaging for assessment of component alignment. Postoperative CT was performed 2 weeks after surgery. Helical CT was performed from the hip to the ankle with a 1-mm slice interval in all cases.

### Preoperative 3D-CT planning

The preoperative CT data were imported into Zed Knee System (LEXI Co., Tokyo, Japan), which is a validated CT-based three-dimensional (3D) preoperative planning system for TKA^19, 20^. Femoral reference points were plotted on these images to define the mechanical axis in the sagittal and coronal planes, the surgical epicondylar axis (SEA), and the posterior condylar axis (PCA). Tibial reference points were then plotted to define the mechanical axis in the sagittal and coronal planes, as well as the anteroposterior axis from the medial third of the patellar tendon attachment to the middle of the posterior cruciate ligament. In both BCR and PS TKA, the femoral component was placed parallel to the mechanical axis in the coronal plane and to 3° flexion relative to the mechanical axis in the sagittal plane. Rotational alignment of the femoral component was placed parallel to the SEA in the rotational plane. In the case that the SEA was at < 3° relative to the PCA, the femoral component was placed at 3° of external rotation relative to the PCA. The tibial component was placed parallel to the mechanical axis in the coronal plane. In BCR TKA, the tibial component was aligned with the patient’s native posterior tibial slope in the sagittal plane. If the patient’s posterior tibial slope was ≥ 10°, the tibial component was set to 10° in the sagittal plane. In PS TKA, the posterior slope of the tibial component was set to 3° in the sagittal plane. For all TKAs, the tibial component was placed parallel to the anteroposterior axis from the medial third of the patellar tendon attachment to the middle of the posterior cruciate ligament in the rotational plane.

### Surgical procedure

The Vanguard XP system used in this study is a recently developed TKA with a U-shaped symmetrical tibial component that retains the anterior and posterior cruciate ligaments. The anterior portion of the tibial component consists of a broad bar to provide sufficient rotating-beam fatigue strength. The femoral component has a new-generation design with a narrowed and funnel-shaped anterior femoral flange.

All TKAs were performed based on the 3D-CT plan by the mid-vastus approach after inflating the tourniquet to 300 mmHg at the beginning of the procedure. All components were fixed with cement. In BCR TKA, distal femoral and proximal tibial osteotomies were performed using an accelerometer-based portable navigation system. The anterior and posterior cruciate ligaments were protected during femoral preparation and the tibia was prepared using a guide designed to preserve a bone island for anterior cruciate ligament insertion. In PS TKA, a balanced gap technique was performed using an image-free navigation system. Rotation of the femoral component was determined according to the navigation system after soft tissue balancing. The patella was resurfaced during all TKAs. The thickness of patellar resection was determined by the thickness of the patellar component to be used. On the first day after TKA, patients were allowed to walk with full weight bearing after the drainage tube had been removed.

### Postoperative evaluation

The postoperative CT data were imported into Zed Knee System (LEXI Co.). The reference points defined on preoperative CT were then transferred to the postoperative CT by matching the pre- and postoperative CT images. The component positions and rotations were evaluated using the common preoperative reference points.

Pre- and postoperative rotational mismatches were evaluated for each of the femorotibial bones and the femorotibial components, using a validated method that has been described previously^11, 12^. The baseline of the femur was defined as the SEA from the lateral epicondylar prominence to the lowest point of the medial sulcus of the medial epicondyle. The baseline of the tibia was defined as the anteroposterior axis from the medial third of the patellar tendon attachment to the middle of the posterior cruciate ligament. Pre- and postoperative rotational mismatch between the femorotibial bones was measured as the angle between the SEA and a line perpendicular to the anteroposterior axis. A positive value of rotational mismatch indicated an externally rotated position of the tibia relative to the femur. Pre- and postoperative rotational mismatch of the femorotibial components was measured as the angle between the center line of the femoral component and the center line of the tibial component in the femoral component plane. A positive value of rotational mismatch indicated an externally rotated position of the tibial component relative to the femoral component. Additionally, the clinical outcomes of BCR TKA and PS TKA were evaluated using a Numerical Rating Scale (NRS) ranging from 0 (no pain) to 10 (severe pain) at one year postoperatively.

### Statistical analysis

Statistical analyses were performed with EZR (Saitama Medical Center, Jichi Medical University, Saitama, Japan), a graphical user interface for R (The R Foundation for Statistical Computing, Vienna, Austria). The patient demographics were compared between BCR TKA and PS TKA using Mann–Whitney U test and Fisher's exact test. The mean values of pre- and postoperative rotational mismatches between BCR TKA and PS TKA were compared using Mann–Whitney U test. The percentage of pre- and postoperative rotational mismatch within ± 5° was compared between BCR TKA and PS TKA using Fisher's exact test. Correlations between pre- and postoperative rotational mismatches of the femorotibial components and bones in BCR TKA and PS TKA were analyzed by the nonparametric Spearman’s rank correlation coefficient. Furthermore, postoperative NRS scores were compared between BCR TKA and PS TKA using Mann–Whitney U test. Correlations between postoperative rotational mismatch and postoperative NRS scores in BCR TKA and PS TKA were analyzed using the nonparametric Spearman’s rank correlation coefficient. Values of *p* < 0.05 were considered statistically significant. A power analysis was performed with G*Power version 3.1.9 (University of Kiel, Germany). A prior power analysis showed that the minimum sample size required to achieve a modest correlation between pre- and postoperative rotational mismatches was 34 knees when using a power of 0.80 and alpha error of 0.05.

### Ethics approval and consent to participate

This study was approved by Institutional Review Board at Mie University hospital (H2018-083). All patients gave informed consent for the use of their surgical data in the study. The study was carried out in accordance with the principles of the Helsinki declaration.

## Results

**Figure 1:**
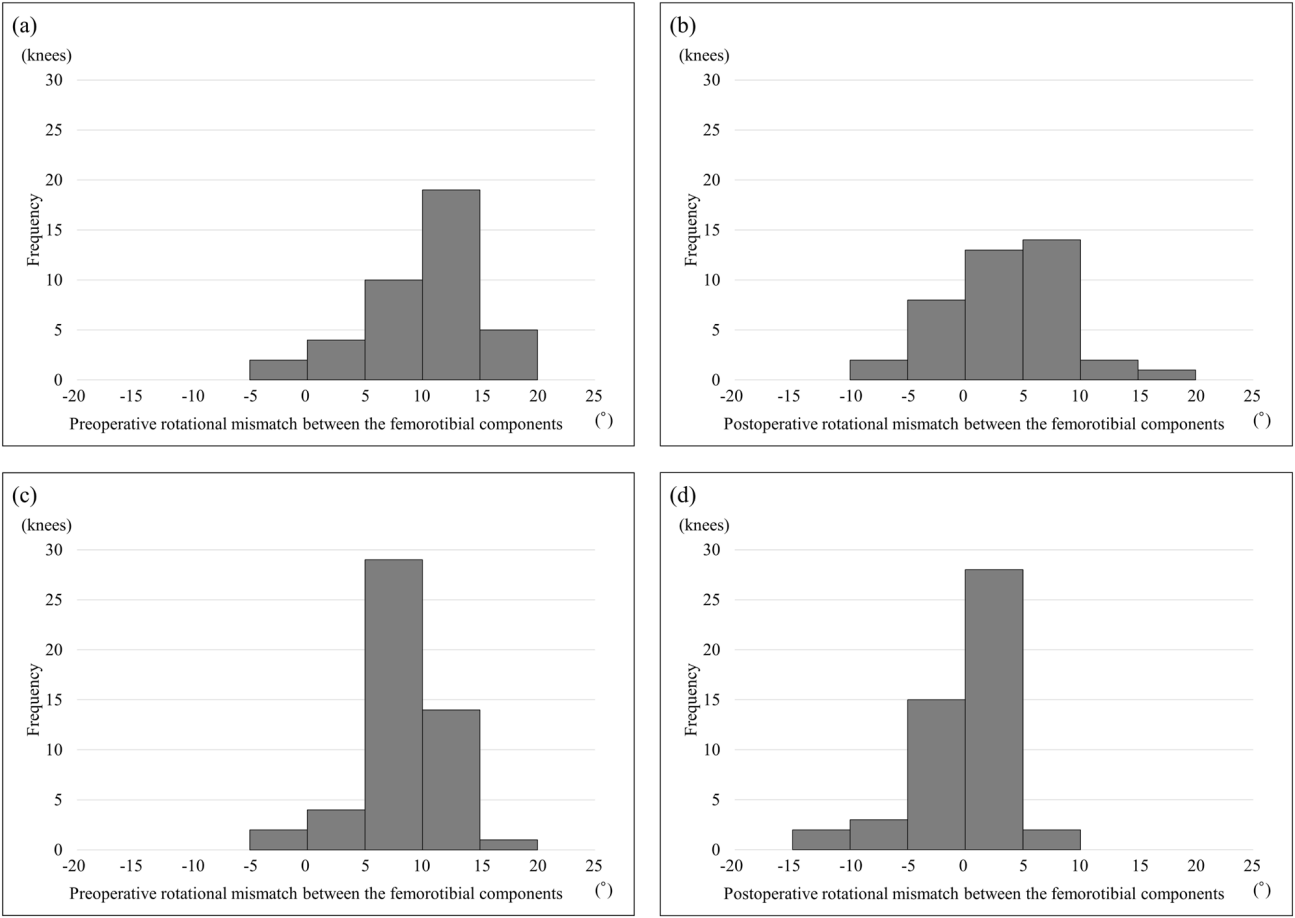
Histograms of pre- and postoperative rotational mismatches between the femorotibial components. Preoperative (**a**) and postoperative (**b**) rotational mismatches for BCR TKA. Preoperative (**c**) and postoperative (**d**) rotational mismatches for PS TKA.

**Figure 2:**
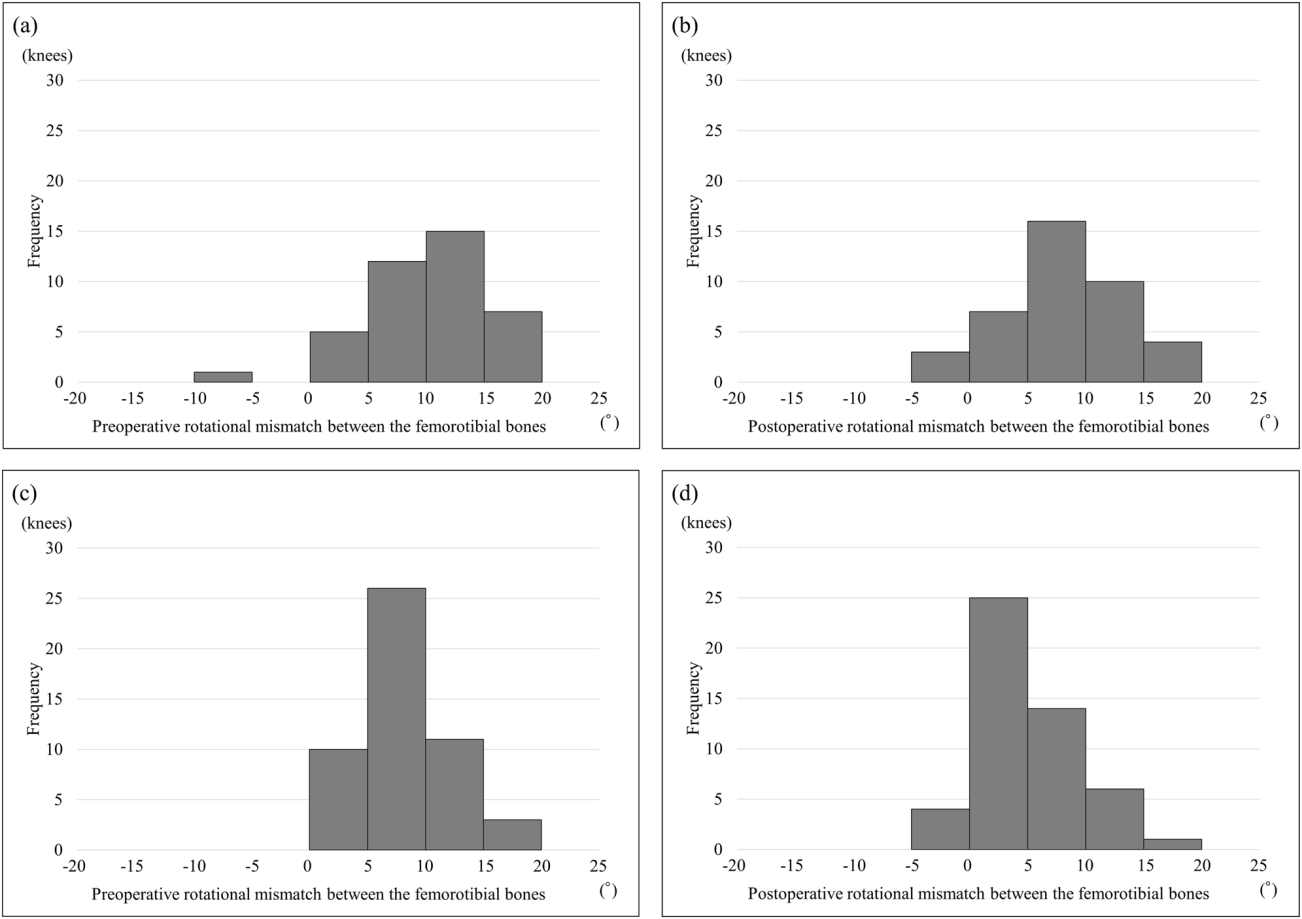
Histograms of pre- and postoperative rotational mismatches between the femorotibial bones. Preoperative (**a**) and postoperative (**b**) rotational mismatches for BCR TKA. Preoperative (**c**) and postoperative (**d**) rotational mismatches for PS TKA.

**Figure 3:**
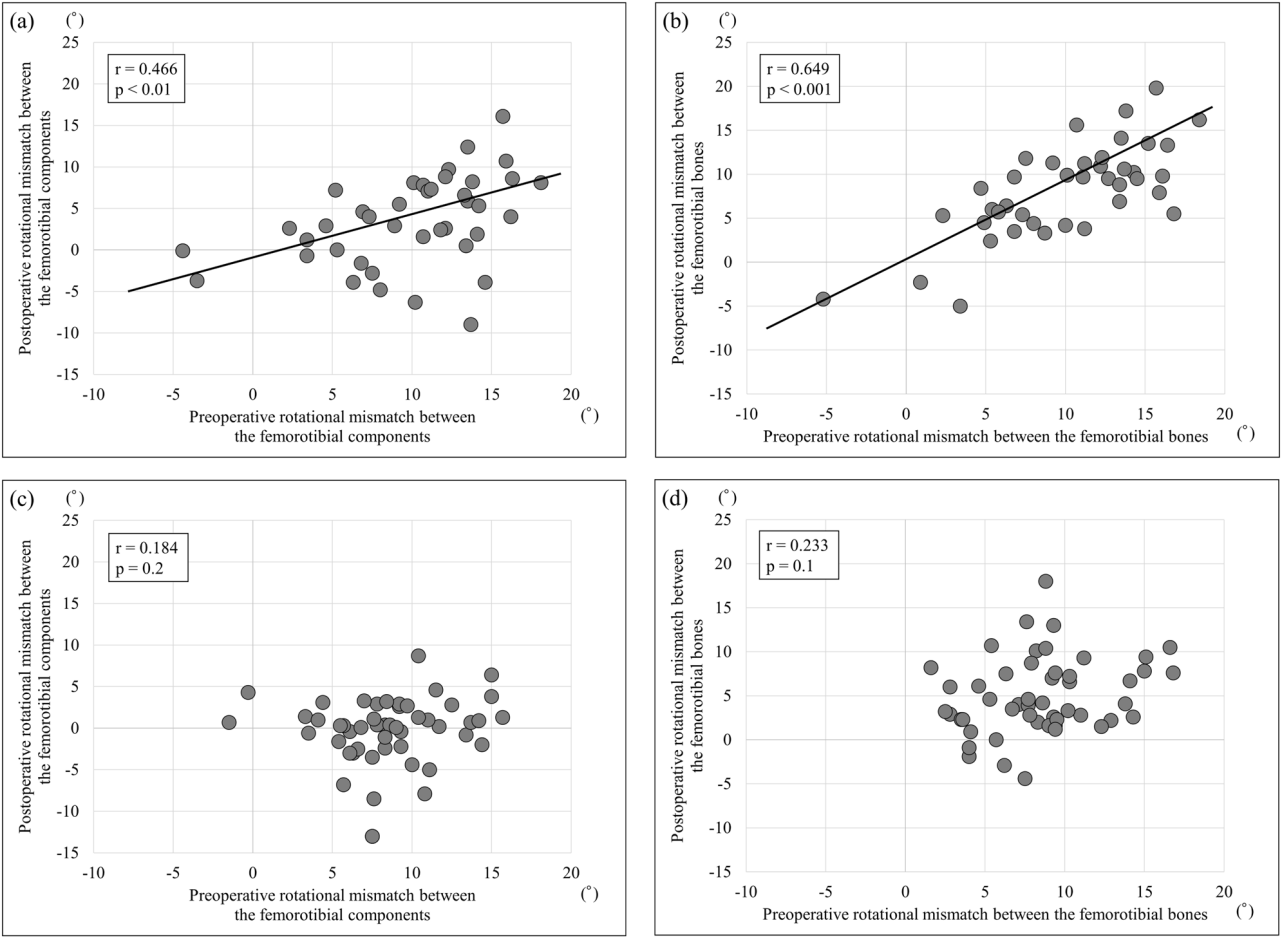
Distributions between pre- and postoperative rotational mismatches of the femorotibial components and bones. A significant positive correlation was found between pre- and postoperative rotational mismatches of the femorotibial components (**a**) and bones (**b**) in BCR TKA. No correlation was found between pre- and postoperative rotational mismatches of the femorotibial components (**c**) or bones (**d**) in PS TKA.

The patient demographics are listed in Table 1. Mean age at operation was significantly higher for BCR TKA than for PS TKA. Mean body mass index was significantly lower for BCR TKA than for PS TKA. Table 2 shows the results of pre- and postoperative image evaluation. No significant differences were found between BCR TKA and PS TKA regarding the mean value of preoperative rotational mismatch between the femorotibial components or that between the bones. The mean value of postoperative rotational mismatch between the femorotibial components and that between the bones was significantly greater for BCR TKA than for PS TKA. The frequency of pre- and postoperative rotational mismatches between the femorotibial components and between the femorotibial bones are shown in Figs. 1 and 2, respectively, for each of BCR TKA and PS TKA. Preoperative rotational mismatch of the femorotibial bones within ± 5° was present in 5 knees (12.5%) in BCR TKA and in 10 knees (20.0%) in PS TKA. Postoperative rotational mismatch of the femorotibial bones within ± 5° was present in 10 knees (25.0%) in BCR TKA and in 36 knees (72.0%) in PS TKA. Preoperative rotational mismatch of the femorotibial components within ± 5° was present in 6 knees (15.0%) in BCR TKA and in 15 knees (30.0%) in PS TKA. Postoperative rotational mismatch of the femorotibial components within ± 5° was present in 21 knees (52.5%) in BCR TKA and in 43 knees (86.0%) in PS TKA. There was no significant difference in preoperative rotational mismatch within ± 5° between BCR TKA and PS TKA, whereas the BCR TKA values were significantly lower than those of PS TKA for postoperative rotational mismatch within ± 5°. Figure 3 shows the distributions between pre- and postoperative rotational mismatches of the femorotibial components and bones in BCR TKA and PS TKA. No correlation was found between pre- and postoperative rotational mismatches of the femorotibial components or those of the femorotibial bones in PS TKA. Preoperative rotational mismatch of the femorotibial components was positively correlated with postoperative rotational mismatch of the femorotibial components in BCR TKA (r = 0.466, p < 0.01). Preoperative rotational mismatch of the femorotibial bones was positively correlated with postoperative rotational mismatch of the femorotibial bones in BCR TKA (r = 0.649, p < 0.001).

**Table 1:** Patient demographics.

	BCR TKA	PS TKA	p value
Age at operation, years	75.5 ± 8.0	72.1 ± 7.9	< 0.05*
Gender (male:female)	5:35	4:46	ns
Body mass index, kg/m^2^	24.8 ± 2.6	28.0 ± 4.8	< 0.01*
Knee flexion angle, °	129.1 ± 11.9	122.1 ± 15.5	ns
Knee extension angle, °	7.6 ± 5.2	7.3 ± 5.4	ns
K–L grade (3:4)	19:21	19:31	ns
Preoperative hip-knee-ankle angle, °	− 9.1 ± 4.0	− 10.1 ± 4.9	ns

*Significant difference: p < 0.05.

**Table 2:** Mean pre- and postoperative rotational mismatch of the femorotibial component and the femorotibial bones for BCR TKA and PS TKA.

	BCR TKA group	PS TKA group	p value
Mean preoperative rotational mismatch between the femorotibial bones	10.0 ± 5.1 (− 5.2 to 18.4)	8.5 ± 3.8 (1.6 to 16.8)	ns
Mean postoperative rotational mismatch between the femorotibial bones	8.2 ± 5.4 (− 5 to 19.8)	5.0 ± 4.4 (− 4.4 to 18)	< 0.001*
Mean preoperative rotational mismatch between the femorotibial components	9.9 ± 5.1 (− 4.4 to 18.1)	8.5 ± 3.6 (− 1.5 to 15.7)	ns
Mean postoperative rotational mismatch between the femorotibial components	3.4 ± 5.4 (− 9 to 16.1)	− 0.4 ± 4.2 (− 13.0 to 8.7)	< 0.001*

*Significant difference: p < 0.05.

The mean values of postoperative NRS scores at one year were 1.48 for BCR TKA and 1.51 for PS TKA. No significant difference was observed between BCR TKA and PS TKA for the mean values of postoperative NRS scores at one year. In BCR TKA, postoperative rotational mismatch of the femorotibial components was correlated with postoperative NRS scores at one year (r = 0.51, p < 0.001). Moreover, postoperative rotational mismatch of the femorotibial bones was correlated with postoperative NRS scores at one year (r = 0.363, p < 0.01). However, no correlation was found between postoperative rotational mismatch of the femorotibial components or those of the femorotibial bones and postoperative NRS scores in PS TKA at one year.

## Discussion

Anteroposterior radiographs can be used to evaluate component and limb alignment in the coronal and sagittal planes^8, 21, 22^. However, evaluation of rotational alignment is difficult on an anteroposterior radiograph. Therefore, CT is commonly used to evaluate rotational alignment^1, 2, 23, 24^. Three-dimensional CT bone models enable more detailed evaluation of alignment as well as rotational mismatch between the femorotibial components and between the femorotibial bones^11, 12^. To the best of our knowledge, the present study is the first to compare pre- and postoperative rotational mismatches between BCR TKA and PS TKA.

The main finding of this study was the positive correlation between pre- and postoperative rotational mismatches of the femorotibial components and also of the femorotibial bones in BCR TKA, which was not found in PS TKA. In other words, preoperative rotational mismatch affected postoperative rotational mismatch in BCR TKA but preoperative rotational mismatch did not affect postoperative rotational mismatch in PS TKA, which is a very interesting result. A functionally intact ACL is a prerequisite for implantation of a BCR TKA device and may contribute to patient satisfaction when retained in TKA^25^. A systematic literature review found that based on intraoperative assessment of the ligament, the ACL is macroscopically intact in more than half of patients with knee OA who undergo TKA, which suggests that BCR TKA could be considered as an alternative to traditional TKA in a large number of patients who undergo this procedure^26^. However, Lavoie et al. have reported a greater frequency and magnitude of postoperative stiffening following BCR TKA compared with PS TKA^27^. Moreover, formation of a cyclops lesion in the anterior compartment, usually after anterior cruciate ligament reconstruction, has been reported following BCR TKA^28, 29^. In addition, Christensen et al. have reported a higher revision rate for BCR TKA with novel prosthesis design compared with conventional TKA, for which the primary failure mechanisms were tibial loosening, ACL impingement, and pain^30^. However, these causes of failure are unclear.

**Figure 4:**
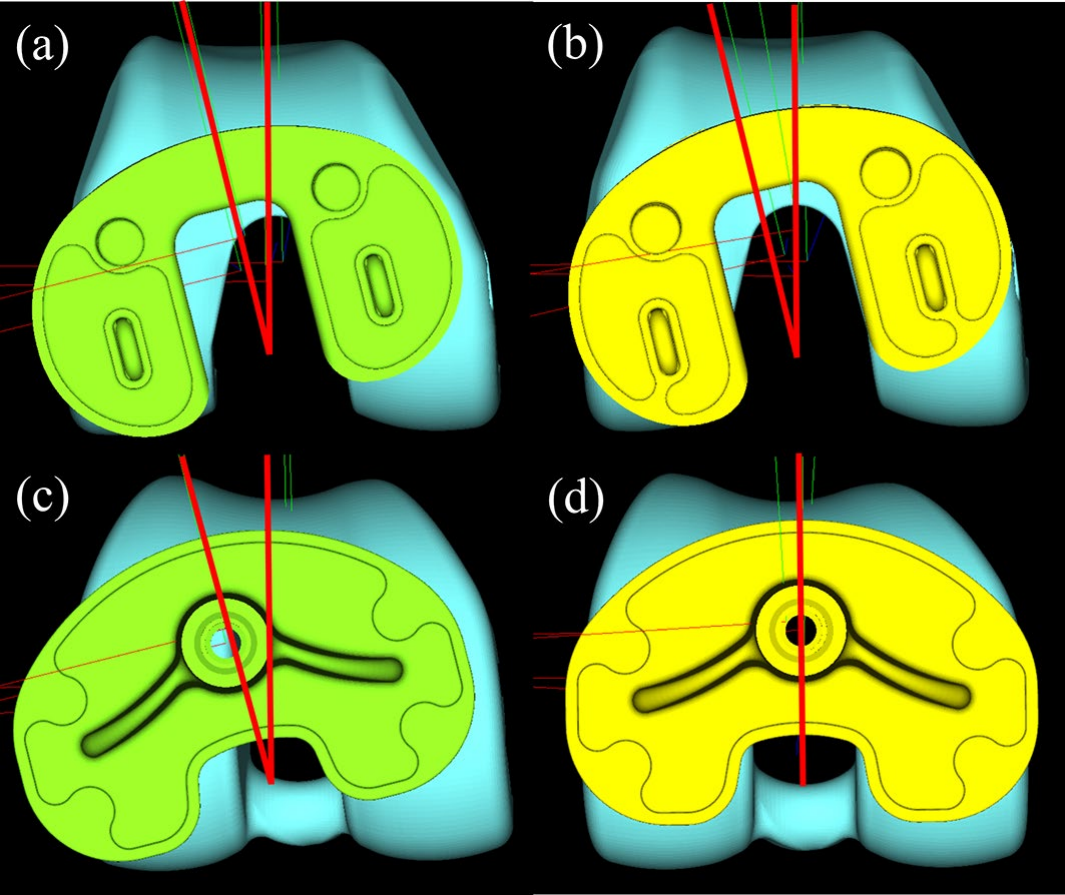
Schema of pre- and postoperative rotational mismatches between the femorotibial components in BCR TKA and PS TKA. In BCR TKA, rotational mismatch observed preoperatively (**a**) remained postoperatively (**b**). In PS TKA, rotational mismatch observed preoperatively (**c**) improved postoperatively (**d**).

In the present study, the rates of postoperative rotational mismatch within ± 5° between the femorotibial components and between the femorotibial bones were significantly lower for BCR TKA than for PS TKA. Furthermore, there was a significant correlation between pre- and postoperative rotational mismatches of the femorotibial bones in BCR TKA. The difference in results between BCR TKA and PS TKA can be attributed to the retention of both cruciate ligaments in BCR TKA, which may have the effect of reducing rotational permittance of the components and bones. These results suggest that in BCR TKA, in which both cruciate ligaments are retained, postoperative rotational mismatch depends on preoperative rotational mismatch; whereas in PS TKA, in which both cruciate ligaments are not retained, postoperative rotational mismatch depends on the tolerance of the components (Fig. 4).

Several recent studies have reported an association of postoperative rotational mismatch with poor clinical outcome. In the present study, rotational mismatches of the femorotibial components and bones were associated with worse early clinical outcomes in BCR TKA. Therefore, it is important to consider preoperative rotational mismatch between the femorotibial components and the bones when the rotational alignments of the femoral and tibial components are determined in BCR TKA.

There were several limitations in this study. First, the sample size was small. Second, CT delivers a higher radiation dose to the patient compared with conventional radiography^31^. Increased radiation exposure has been related to increased risk of various cancers, indicating the importance to minimize radiation exposure as much as possible^32^. However, the present results confirm the usefulness of CT for evaluation of the rotational alignment of the components, which is difficult to achieve with conventional radiography. Third, rotational mismatches were evaluated using CT in the supine position without weight bearing. Conventional CT scans are performed with patient in supine position, with the knee fully extended and the lower limb muscles relaxed, thus far from the physiological weight-bearing conditions. It is known that quadriceps muscular contraction and knee flexion which occur in weight-bearing influence patellar stability and alignment^33^. Therefore, it is unclear whether the same results would be obtained in the standing position with weight bearing. Future studies on rotational mismatches of the femorotibial components and bones in weight bearing CT are also needed.

In conclusion, we evaluated the relationship between pre- and postoperative rotational mismatches of the femorotibial components and bones in BCR TKA and PS TKA. In contrast to PS TKA, BCR TKA showed a positive correlation between pre- and postoperative rotational mismatches of the femorotibial components and bones. These results suggest that preoperative rotational mismatch should be used as a reference when rotational alignments of the femoral and tibial components are determined in BCR TKA.

## Data Availability

The datasets used and/or analysed during the current study available from the corresponding author on reasonable request.
